# Rolling back malaria

**DOI:** 10.7554/eLife.07364

**Published:** 2015-03-30

**Authors:** Philippa C Matthews

**Affiliations:** NIHR Academic Clinical Lecturer in the Nuffield Department of Medicine, University of Oxford, Oxford, United Kingdomp.matthews@doctors.org.uk

**Keywords:** science writing competition, malaria, outreach, open access

## Abstract

Efforts to fight malaria in Africa are proving successful in many countries, but a population explosion means that there is still a long way to go.

This article by Philippa C Matthews (pictured) was the winning entry in the 2015 Access to Understanding science-writing competition organized by the British Library in partnership with eLife and Europe PMC. Access to Understanding promotes understanding of biomedical research. Competition entrants were challenged to summarize selected scientific research articles in plain English, explaining why the research was done, what was done and why it is important.

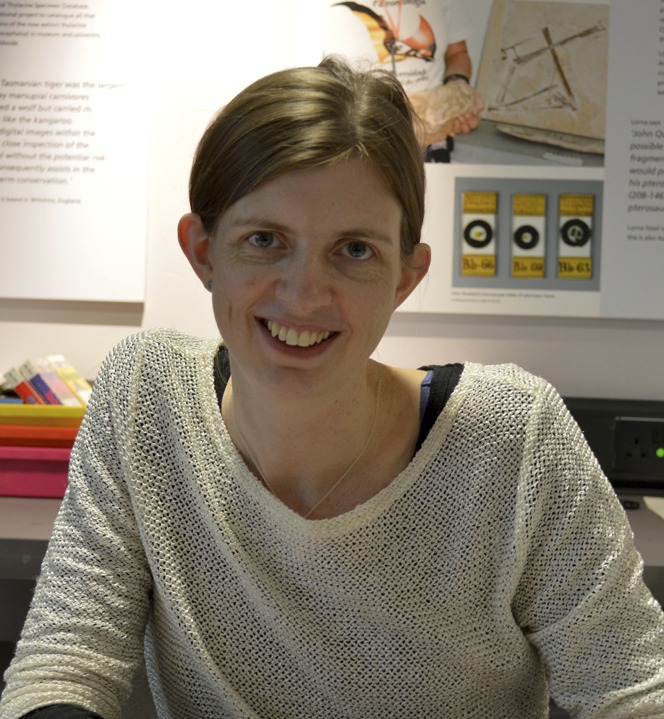

*Plasmodium falciparum* is the most deadly of all malaria parasites. Children are particularly vulnerable to the devastating consequences of this infection, and the World Health Organisation (WHO) estimates that a child in Africa dies from malaria every minute (WHO, 2014). In recognition of this crisis, malaria has become a headline priority in global health, with campaigns such as the Roll Back Malaria initiative spearheading a huge international effort to tackle the disease.

Abdisalan Noor and colleagues set out to assess how much progress has been made across Africa in the Roll Back Malaria era. Have the immense resources deployed made a real difference to some of the world's most vulnerable populations? How has the burden of malaria altered in the decade since 2000? And can we identify whether infection risks have changed by country or region? With these questions in mind, Noor and colleagues developed a method to measure the changing patterns of malaria risk in Africa as precisely as possible (Noor et al., 2014).

The symptoms of malaria can mimic those of many other infections, and suspected cases of malaria are often treated in the absence of a definite diagnosis and without attending a hospital or clinic. For these reasons, trying to get a clear picture of the scale of the malaria problem in Africa is a major challenge. Noor and colleagues chose to measure malaria by the most accurate method available, which involved looking for studies that had actually identified *P. falciparum* parasites in the blood. Their final analysis draws on data collected from 3.5 million individuals from over 26,000 surveys spanning 49 regions of Africa, with each piece of information linked to its precise geographic origin by satellite technology.

The researchers—who are based at the Kenya Medical Research Institute-Wellcome Trust Research Programme in Nairobi, Oxford University and the WHO Regional Office for Africa in the Republic of Congo—fed this vast mine of data into a carefully constructed computational analysis. Each piece of information was adjusted to account for when and where it was collected, and the data were then standardized to work out the rate of infection in the age group of interest—the highly vulnerable population of children aged 2–10 years. They also factored in a host of complex influences on malaria transmission, such as urbanisation and climate. The final output was a measure of malaria risk for each individual square kilometre of Africa, first in 2000 and again a decade later. Each of these tiny squares was then classified into one of eight different malaria risk categories.

From this analysis, Noor and colleagues report several substantial and encouraging improvements in the patterns of malaria in Africa. Strikingly, they calculated that 217 million people in Africa were living in a lower risk area in 2010 than they did in 2000. They also found an overall reduction in malaria transmission in 40 of the 44 countries that they assessed in detail. Moreover, four territories—South Africa, Eritrea, Ethiopia and Cape Verde—had successfully reduced malaria into the lowest risk category by 2010. By this time, the majority of the transmission in the highest risk category was occurring in just ten countries.

However, the results also provide a sobering insight into the effects of the population explosion in Africa. This has increased the total numbers of people at risk of malaria; worryingly, over 50% of the population still live in regions of substantial risk. Rates of infection have also remained unchanged or have increased in some countries between 2000 and 2010: Malawi and South Sudan are highlighted as areas for increasing concern, and high transmission has continued across many parts of Nigeria and the Democratic Republic of Congo.

Noor and colleagues also report some areas of difficulty. Despite a data collection effort that spanned eight years, they were still unable to find enough information to assess the malaria risk for certain regions of Africa. Moreover, the vastly complex nature of malaria transmission cannot be completely captured or measured by a computer-based method. Overall, however, the results of this analysis provide valuable feedback for organizations trying to improve population health in many parts of Africa.

As well as amassing a vast amount of information about the distribution of malaria, the researchers also thought hard about the impact of the rapidly changing social and geographical landscape of Africa, ranging from urbanisation to changes in rainfall patterns. The detailed maps they have produced also highlight how the risks of an infection can wax and wane, reminding us of the need to constantly re-assess the best way to keep pace with changing patterns of disease. Noor and colleagues conclude with cautious optimism about the progress made since 2000, but provide a timely warning that the challenge of malaria is far from over.
